# Supplementary material for: Comparative Efficacy of 14-Day Tegoprazan-Based Triple vs. 10-Day Tegoprazan-Based Concomitant Therapy for Helicobacter pylori Eradication
Source: J Pers Med. 2022 Nov 17;12(11):1918. doi: 10.3390/jpm12111918 (PMC9695183; doi:10.3390/jpm12111918)
Supplement: Supplementary file 1 [file jpm-12-01918-s001.zip › 04. TAC14 vs TACM10-Table S1_revision (22.11.9).pdf]

Table S1. Adherence and adverse events of second-line *H. pylori* eradication therapy

	14-day rabeprazole-based PBMT therapy (N=21)	14-day tegoprazan-based PBMT therapy (N=71)	*P*-value
**Adherence,[a] n (%)**	18 (85.7)	60 (84.5)	> 0.999
Loss of follow-up	1 (4.8)	8 (11.3)	0.679
Insufficient medication	2 (9.5)	3 (4.2)	0.320
**Adverse event,[b] n (%)**			
Any adverse event	13 (61.9)	32 (45.1)	0.175
Mild	11 (52.4)	29 (40.8)	
Moderate	1 (4.8)	3 (4.2)	
Severe	1 (4.8)	0 (0.0)	
General weakness	1 (4.8)	1 (1.4)	0.406
Dizziness	1 (4.8)	1 (1.4)	0.406
Hedache	0 (0.0)	1 (1.4)	> 0.999
Myalgia	1 (4.8)	1 (1.4)	0.406
Acid regurgitation	0 (0.0)	0 (0.0)	N/A[d]
Nausea or vomiting	8 (38.1)	17 (23.9)	0.200
Dysgeusia	2 (9.5)	8 (11.3)	> 0.999
Abdominal discomfort	1 (4.8)	6 (8.5)	> 0.999
Abdominal pain	0 (0.0)	1 (1.4)	>0.999
Diarrhea	2 (9.5)	11 (15.5)	0.725
Constipation	0 (0.0)	0 (0.0)	N/A[d]
Skin rash	2 (9.5)	0 (0.0)	0.050
Others[c]	2 (9.5)	3 (4.2)	0.320

[a]Adherence is determined as administration of ≥ 80% of prescribed medications.

[b]Percentage is calculated based on the ITT population.

[c]Other adverse events include dry mouth and sweating.

[d]N/A indicates that *P*-value cannot be calculated because the number of events in both groups is zero.

PBMT indicates bismuth-containing quadruple therapy comprising a PPI or P-CAB, bismuth, metronidazole, and tetracycline.

ITT, intention-to-treat; PP, per protocol; PPI, proton pump inhibitor; P-CAB, potassium-competitive acid blocker; N/A, not applicable.
