# Supplementary figures and images for: Comparative Efficacy of 14-Day Tegoprazan-Based Triple vs. 10-Day Tegoprazan-Based Concomitant Therapy for Helicobacter pylori Eradication
Source: J Pers Med. 2022 Nov 17;12(11):1918. doi: 10.3390/jpm12111918 (PMC9695183; doi:10.3390/jpm12111918)

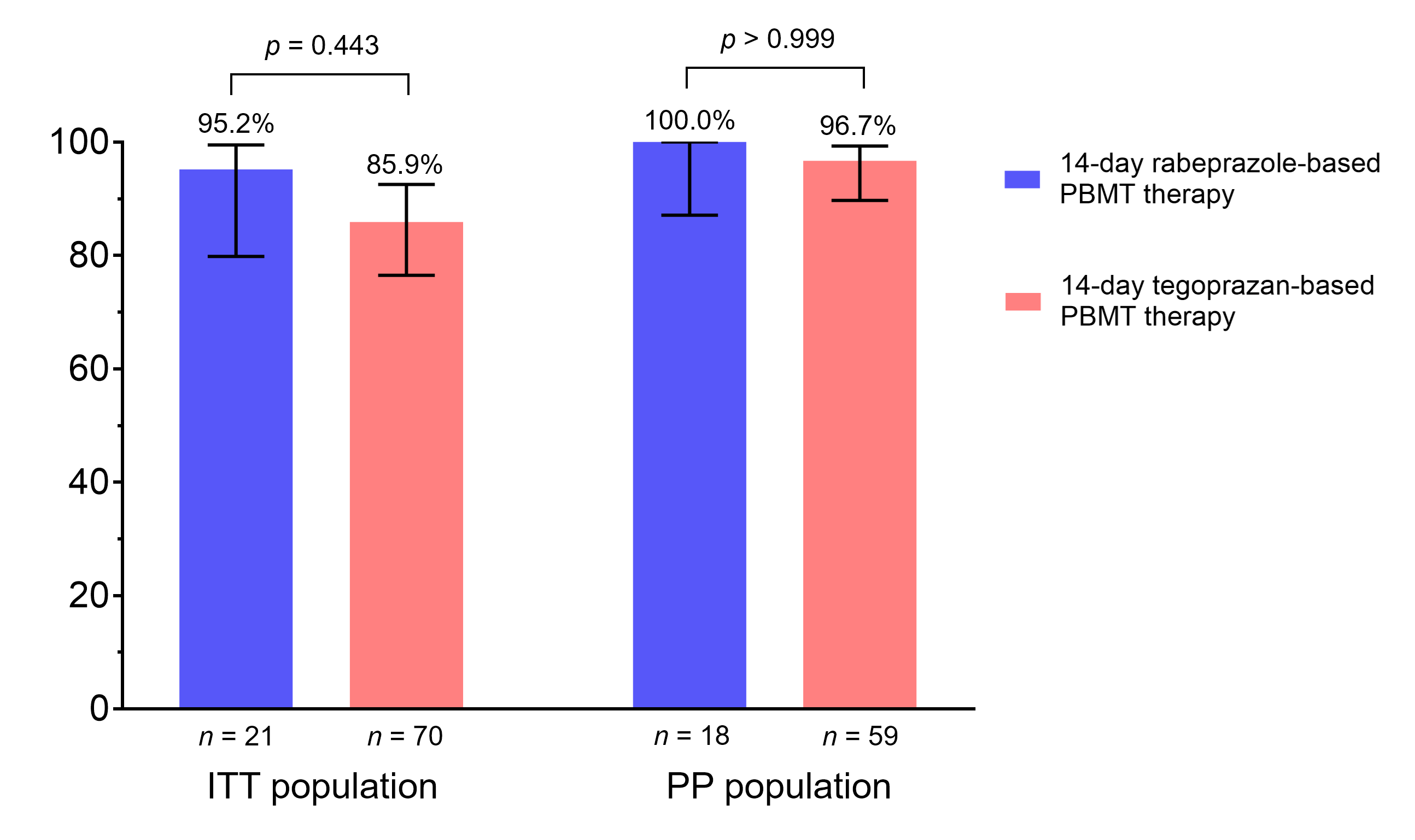

Supplement: Supplementary file 1 [file jpm-12-01918-s001.zip › Figure S1_revision(22.11.16).tif]
